# Advantages and Pitfalls of Mass Spectrometry Based Metabolome Profiling in Systems Biology

**DOI:** 10.3390/ijms17050632

**Published:** 2016-04-27

**Authors:** Ina Aretz, David Meierhofer

**Affiliations:** Max Planck Institute for Molecular Genetics, Ihnestraße 63-73, 14195 Berlin, Germany; gielisch@molgen.mpg.de

**Keywords:** metabolome profiling, mass spectrometry, nuclear magnetic resonance (NMR) spectroscopy, systems biology, multiple reaction monitoring (MRM), LC-MS, derivatization

## Abstract

Mass spectrometry-based metabolome profiling became the method of choice in systems biology approaches and aims to enhance biological understanding of complex biological systems. Genomics, transcriptomics, and proteomics are well established technologies and are commonly used by many scientists. In comparison, metabolomics is an emerging field and has not reached such high-throughput, routine and coverage than other omics technologies. Nevertheless, substantial improvements were achieved during the last years. Integrated data derived from multi-omics approaches will provide a deeper understanding of entire biological systems. Metabolome profiling is mainly hampered by its diversity, variation of metabolite concentration by several orders of magnitude and biological data interpretation. Thus, multiple approaches are required to cover most of the metabolites. No software tool is capable of comprehensively translating all the data into a biologically meaningful context yet. In this review, we discuss the advantages of metabolome profiling and main obstacles limiting progress in systems biology.

## 1. Introduction

Metabolomics offers a framework for analyzing individuals with a specific phenotype at a molecular level in cell biology, personalized medicine, and systems biology [1,2,3]. Metabolites are small molecules participating in metabolic reactions, which are necessary for cellular function, maintenance and growth [4]. Typically, metabolites range from 50 to 1500 Da, while their concentrations span several orders of magnitude. The metabolome is highly dynamic, time-dependent, and metabolites are sensitive to many environmental conditions. It is still unknown how many different metabolite species exist within a cell or organism; predicted are at least 2000 in mammals [5], the Human Metabolome Database (HMDB) [6] currently has more than 8000 verified entries. Plants and bacteria outnumber the diversity of compounds by two orders of magnitude; more than 200,000 metabolites are estimated, for example, to exist in the plant kingdom [7,8]. Metabolites are extensively exchanged with the environment, e.g., food intake, excretion, inhalation, secondary metabolites, such as medications, flavorings, and recreational drugs, which can be further processed by the gut microbiome or organs. Furthermore, metabolites are, chemically, very diverse (polarity, charge, pKa, solubility, volatility, stability, and reactivity), consequently no single method can capture and analyze the entire metabolome at once. Hence, many extraction methods were developed to identify and quantify specific classes of metabolites. The most commonly used approaches to explore the metabolome are gas chromatography (GC), liquid chromatography (LC) or, to a lesser extent, capillary electrophoresis (CE), online coupled to a mass spectrometer (MS), as well as nuclear magnetic resonance (NMR) spectroscopy [9]. Nevertheless, the contextual and chemical diversity makes the detection of a whole set of metabolites present in a biological sample an ambitious goal [10,11].

Systems biology is a novel subject in the field of life science, which aims to understand biological systems as a whole entity, under a variety of conditions [12]. The availability of huge amounts of quantitative data and the development of computational methodologies are the main reasons for the emergence of systems biology [12]. The complete biological model can only be discovered if different levels of gene expression, proteins, and metabolites are considered in an analysis. Multi-omics data can be combined as predictor variables in order to identify models that predict phenotypes and elucidate biomarkers [13].

Metabolites are regarded as the final response of a biological system to environmental changes or gene regulations and, thus, an aberrant metabolism can be linked to disease severity and phenotypes. The metabolome is, thereby, the most predictive of phenotype [1,4]. Metabolic profiling refers to the comprehensive identification and quantification of metabolites in a biological system [14,15]. Systems biology-based metabolome profiling is a new and promising field, which is still puzzled by several aspects, such as sample preparation, identification, standardization, and translating results into biological meaningful data.

Metabolomics and systems biology-driven studies have a great potential for hypotheses generation and dissection of signaling networks in an unbiased or untargeted fashion [16]. The main advantage of systems biology-based metabolomics is the connection of metabolic networks to the underlying reaction pathway structure [7]. Thus, systems biology based metabolomics has a lot of benefits, such as elucidating connectivity maps of pathways, but is still far from covering the entire metabolome.

In this review, we will focus on the advantages and challenges in metabolome profiling for systems biological approaches and cover various techniques and their limitations with a special spotlight on real-time metabolome profiling as a very promising new strategy.

## 2. Sample Preparation

Typically, a sample preparation protocol contains a solvent extraction step, ultrafiltration, solid-phase extraction and, optionally, a chemical derivatization step [1]. The optimization of sample pretreatment is an important point in method development, since it ensures reproducibility. This review is not focusing on pretreatment strategies and we refer to a variety of other review articles [17,18,19,20].

### 2.1. Derivatization

Derivatization methods apply reagents, which chemically modify and transform a specific target structure. Thus, derivatization is one of the most effective method to improve the detection characteristics of metabolites in GC- or LC-MS, binding to LC columns, or to stabilize compounds. For example, formaldehyde can be used to label the amine groups through reductive amination. Silylation is a common derivatization reagent in GC-MS, as it transfers a wide range of functional groups, even though some products are not very stable [21].

By derivatization, a sensitivity gain by several orders of magnitude can be achieved. The drawbacks are that an additional derivatization step is introduced and not all metabolites can be derivatized by only one reagent. Furthermore, the mass spectra are different in terms of parent and fragment masses from their endogenous counterparts. Thus, databases for endogenous compounds cannot be applied for reference matching and derivatization-specific databases are rare or have to be built from scratch.

A combination of derivatization and internal standards (ISs), named isotope-coded derivatization (ICD), is a good alternative for relative quantification, in order to improve analytical precision, (reviewed in [22,23]). Thereby, one sample is derivatized with a naturally-labeled reagent, while the other sample is separately derivatized with the isotopically-labeled reagent. After differential labelling, the samples are pooled and processed jointly to avoid any method biases. Thus, this method offers new perspectives for quantitative metabolite profiling, where current protocols can easily comprise over a hundred metabolites [22]. ICD is very similar to the classic stable isotope labeling by amino acids in cell culture (SILAC) approach used in proteomics, where specific amino acids are incorporated into newlysynthesized proteins [24]. Thus, ICD works well for selected compounds or compound groups, such as amino acids, glycans, phosphometabolites, amines, nucleotides, carboxylic- and fatty acids [22].

### 2.2. Internal Standards

In mass spectrometry, ISs are the method of choice for absolute or relative quantification, calibration curves, recovery rate determination, or to correct for matrix effects. Typically, ISs are isotopically (^13^C, ^15^N or deuterium)-labeled counterparts of compounds of interest. The ISs have the same chemical behavior as their endogenous counterparts, do not show drastic chromatographic isotope effects, and have the same retention time. The application of ISs is widespread, but one drawback is that they are not 100% labeled. Thus, an additional and indistinguishable unlabeled peak of typically 1%–2% intensity, depending on the impurity ratio may occur in the mass spectrum. Therefore, the amount of introduced ISs should be reduced to a minimum in order to avoid these interferences. Furthermore, isotopically-labeled ISs are not practical for large profiling approaches, since they are expensive and not available for the majority of compounds or it is impossible like for *de novo* identifications. To circumvent this bottleneck, only a few ISs, matching to similar compound groups, are selected. Ionization suppression or enhancement is matrix-specific and shows fluctuations over the elution time. This is not always properly reflected by only using a few ISs.

Alternatives for isotopically-labeled ISs are synthetic compounds, which are very similar to the target compound, such as a 17-carbon chain sphingosine instead of the natural 18-carbon chain sphingosine [25]. Label-free approaches, which aim to determine the relative amount of proteins based on precursor signal intensities or on spectral counting, are well established in proteomics [26]. Several methods have been developed to align chromatographic profiles of non-targeted data without the need of ISs for normalization, like applied in XCMS Online [27,28].

## 3. Data Acquisition and Analysis

### 3.1. GC-, LC-MS, and NMR in Metabolomics

Most metabolic profiling studies are performed using chromatographic separation online coupled to mass spectrometers, usually gas chromatography (GC)-MS and liquid chromatography (LC)-MS, as well as nuclear magnetic resonance (NMR) spectroscopy [29].

NMR is generally accepted as the gold standard in metabolite structural elucidation, due to its high selectivity, analytical reproducibility, non-destructive nature, and simplicity of sample preparation. However, it suffers from relatively low sensitivity compared to MS [30].

Mass spectrometry-based approaches for metabolic profiling have the advantages of a high sensitivity and selectivity, as well as high throughput and depth of coverage [11]. GC-MS has advantages of a greater chromatographic resolution [31,32,33] when compared to LC-MS-based methods, a good retention of small compounds that elute early with the solvent front in reverse-phase LC-MS methods [34], and large spectral libraries [35]. Unfortunately, the thermal stability of the stationary phase, metabolites and their derivatives limit the metabolome coverage derived by GC-MS [11]. Furthermore, several metabolites can just be analyzed by GC-MS after derivatization and this might introduce variability and produce derivatization artifacts [30].

Due to its high sensitivity and wide range of molecules that can be analyzed, the usage of LC-MS has expanded rapidly over the past ten years [1,29].

Bingol and colleagues introduced a strategy, which combines direct infusion MS with NMR (SUMMIT MS/NMR) for the identification of unknown metabolites in complex mixtures [9]. First, the chemical formulas of compounds in the mixture are identified from accurate masses by MS and, subsequently, all possible structures are generated [9]. Second, NMR spectra of each member of the structural manifold are predicted and compared with the experimental spectra to identify the molecular structure that match the information obtained from MS/NMR [9]. With this method, different types of metabolites, such as amino acids, polyamines, nucleic acids, nucleosides, and carbohydrate conjugates could be identified in an *E. coli* extract [9]. This approach opens new possibilities in biomedicine, synthetic chemistry, or food sciences for high-throughput identification of unknown metabolites and to overcome limitations of databases [9]. Thus, it is possible to combine different approaches, but due to instrumentation limitations usually just one analytical method is used [36,37].

### 3.2. Chromatographic Dimension

The metabolome is a collection of compounds with diverse physicochemical properties; no single retention mechanism is adequate to resolve complex sample mixtures that vary widely in their polarity, charge, and stability [1]. Usually, polar and nonpolar metabolites are separated and analyzed in parallel by according analytical columns.

Reversed-phase liquid chromatography (RPLC) is extensively used, because of its reproducible and predictable retention times, wide applicability to various classes of metabolites, and mobile phase compatibility for direct coupling to electrospray ionization mass spectrometry (ESI-MS) [1]. The major disadvantage of RPLC is the poor recovery of hydrophilic compounds. This problem can be solved by the use of hydrophilic interaction chromatography (HILIC), which requires longer re-equilibration and can show retention time drifts [38]. Nevertheless, HILIC is the most commonly used separation mode in LC-MS for polar metabolites that is used together with RPLC for comprehensive metabolomic studies.

The combined use of HILIC and RPLC is an optimal method to increase metabolome coverage, because it is ideal for retaining extremely polar and lipophilic compounds [39]. The main disadvantage is that the applied mobile phases of HILIC and RPLC are incompatible and, thus, two separate runs are necessary, which reduces sample throughput. Furthermore, different chromatographic conditions can cause redundant data analysis and sample preparation is more complicated due to the requirement of different sample buffers [39,40].

To overcome these problems, Haggarty and colleagues applied a method where a sample is injected onto both, RP and HILIC columns in tandem in a single injection [41]. To solve the problem with the solvent strength incompatibility between the columns, a high-aqueous mobile phase at low flow rate for the RPLC column and a high-acetonitrile mobile phase at high flow rate for the HILIC column were used [39].

This tandem column approach was already successfully applied with quadrupole, time of flight (TOF), and orbitrap mass analyzers [39,41,42,43]. This method could significantly increase the amount of identified metabolites in a single analytical run, but one disadvantage is that isomers cannot be separated [39].

### 3.3. Non-Targeted, Targeted, and Real-Time Metabolome Profiling

Experimental approaches can be classified into non-targeted and targeted metabolomics. Non-targeted metabolomics approaches are widely used for *de novo* analyte identification, where no prior knowledge about compounds is necessary. In contrast, targeted metabolomics aims to detect *a priori* selected analytes, on the basis of known parent and fragment masses, with a method termed multiple reaction monitoring (MRM). Once optimized for every transition, the MRM approach has the advantage of selective and sensitive measurement of the selected analytes in complex samples. The specificity is achieved by fragmenting the analyte and by monitoring both parent and one [44,45,46,47,48] or more product ions simultaneously [49,50,51,52,53].

Savolainen and colleagues developed a GC-MS method using a high scan speed (20,000 Da/s), which combines targeted and non-targeted metabolomics into one single accurate, reproducible, and reliable method [34]. This method has the advantage of measuring predefined metabolites relevant to the study question and, additionally, identifies unexpected metabolites [34].

Direct sample injection on high-resolution mass spectrometers is an effective way to maximize analytical throughput, since sampling, sample preparation, and measurements are time-intensive and difficult to automate [54,55]. The applicability of direct injection in metabolomics is extended by advanced instrumentation capable of high-resolution, accurate mass measurements and tandem MS (such as FT-ICR-MS and orbitrap MS) [30]. Flow injection analysis/mass spectrometry (FIA/MS), a method using a HPLC without a column, was applied for metabolite fingerprinting [56], screening in drug discovery [57] and detection of pesticides in food [58]. Matrix effects resulting in inaccurate identification and quantification are the main disadvantages of direct sample injection.

Fuhrer and colleagues established a robust platform for high-throughput, accurate mass, and non-targeted metabolome profiling of *E. coli* extracts [54]. This method is ideal for initial untargeted metabolome screens where a variety of samples need to be analyzed rapidly [54].

Link and colleagues went a step further and performed real-time metabolome profiling by direct injection of living bacteria, yeast, and mammalian cells into a Q-TOF MS in order to reveal metabolic switches between starvation and growth [55]. They detected 10,466 ions with distinct mass-to-charge ratios, which could be assigned to around 300 metabolites in 15–30 second cycles. The real-time data were confirmed by comparison to a manual sampling and extraction method by flow-injection TOF-MS. By applying the real-time method, more time points were analyzed, more metabolites were identified, and detected ion intensities were higher than those derived from manually-obtained samples [55]. Thus, metabolome profiling of living cells can be monitored in real-time over extended periods to follow the dynamics of metabolic processes. Furthermore, the consequences of any kind of cell manipulation can be studied in real-time, leading to new experimental approaches that could not be performed before.

### 3.4. Metabolic Flux Analysis

The original method of metabolic flux analysis (MFA) studies systems at a metabolic steady state, by determining metabolite transport rates within and out of tissues, cells, or their sub-compartments, which are then balanced in reaction networks to provide estimates of intracellular fluxes [59]. The limitation of this classical MFA approach is that it cannot resolve fluxes through parallel pathways, such as glycolysis and pentose phosphate pathway, circular pathways or reversible reactions [60]. To overcome the limitation in MFA, isotope ^13^C tracers, such as the main substrates for mammalian cell culture, glucose and glutamine, can be used [60]. Labeled nutrients in biological systems spread through the network as a function of metabolic activity and produces labeling patterns in the backbone of metabolic intermediates over time [59]. *In vivo* fluxes are calculated by mathematical models that describe the label propagation from isotopic patterns and extracellular transport rates [59].

Isotope labeled-MFA of co-cultures can be performed, in order to determine inter-species metabolite exchange and population dynamics. This method simplifies experimental and analytical procedures for co-culture flux analysis, improves accuracy, and allows flux elucidation in systems where physical separation of cells or proteins is difficult [61].

Recently, we applied an isotope based flux analysis in order to determine the enzymatic activity of mutated pyrroline-5-carboxylate synthase (P5CS), a protein of the mitochondrial proline cycle. The mutation causes an autosomal-dominant form of cutis laxa with progeroid features. The flux analysis revealed that mutated cells had a reduced P5CS enzymatic activity leading to a delayed proline accumulation [62].

The advantage of fluxomics is that the turnover of virtually any metabolite can be studied by isotope labeling. New pathways or disease-causing genes can be elucidated, a perfect method for specific tasks. Systems biological approaches are mainly hampered by the complexity of the entire system, as most pathways are not straightly leading from A to B, introducing a bias and are, hence, difficult to compute.

### 3.5. Mass Spectrometry Imaging

Mass spectrometry imaging (MSI) is a spatially-resolved label-free technique, which can directly identify and map the spatial distribution and abundance of known or unknown molecular species in tissues [63]. Metabolites are the direct measure of metabolic activities and can therefore be correlated to phenotypes. Many critical parameters, such as the discrepancy between the low number of detected metabolites versus the real number of possible metabolites, the bias against compound classes and the overlap of compartmentalized metabolic processes in tissue samples, complicate the interpretation of metabolite profiles [7]. As the two or three dimensional distribution of metabolites in specific tissue parts or even subcellular compartments are crucial for many scientific questions, one option to resolve their precise location is MSI. Conventional imaging techniques are not suitable for simultaneous visualization of endogenous molecules. MSI, using matrix-assisted laser desorption/ionization quadrupole ion trap timeofflight (MALDI-QIT-TOF), was used to visualize the spatial distribution of metabolites in tissue sections [64], or plant surface metabolites [65]. Nanospray desorption electrospray ionization (nano-DESI), combined with MS/MS, enabled simultaneous imaging and identification of a large number of metabolites and lipids with a spatial resolution [66]. Three-dimensional secondary ion mass spectrometry (SIMS) imaging was used to investigate the cellular metabolite uptake on the single cell level [67]. Even MS free systems, such as an electrochemical camera chip for simultaneous imaging of multiple metabolites in biofilms was applied [68].

Limited by the absence of a comprehensive MSI method, normalization, and sample preparation techniques suitable for all metabolite classes, we can barely talk about a systems-wide approach, which covers the metabolome. Derivatization enables increased detection of metabolites that are hard to ionize, but may cause analyte suppression or delocalization [69]. Nevertheless, MSI in all its variants is a valuable diagnostic and systems biology tool, capable of providing comprehensive 2D–3D spatial distribution of selected metabolites, like amino acids, lipids, carnitines, or carbohydrates [63,70] in a wide variety of cells and tissues.

### 3.6. Metabolite Databases

Metabolite databases are repositories of metabolite information, based on experimental confirmation or *in silico* prediction, and usually contain MS/MS data in order to facilitate metabolite identification. The most established repositories for biological compounds are METLIN, featuring 242,000 entries, 14,000 with high-resolution MS/MS data [71], the Human Metabolome Database (HMDB), with more than 40,000 annotated metabolite entries, including “detected” metabolites (those with measured concentrations or experimental confirmation of their existence) and “expected” metabolites (those for which biochemical pathways are known or human intake/exposure is frequent, but the compound has yet to be detected in the body) [6], and MassBank [72].

Several *in silico*-generated compound databases are available as well, such as MINE, containing over 571,000 compounds [73], MyCompoundID, using an evidence-based metabolome library (EML) with 375,809 predicted metabolites [74]. Matching metabolite spectra against such huge *in silico* databases can give rise to too many false-positive identifications just by chance. Based on our experience, databases contain several erroneous entries. Especially, the MS spectra are sometimes of poor quality, they are recorded by different groups with different MS instrumentations, and the provided instrument settings are sometimes very scarce.

All data derived from metabolome profiling studies should be made publically available in according depository databases. Thus, full transparency is guaranteed and datasets can be reused for future identification, quantification, secondary analysis, or systems biology approaches. Data storage, maintenance, and harmonization are critical points, since abandoned or broken depositories would cause a significant loss of knowledge. Currently, just a few metabolomics-specific depositories, such as MetaboLights [75] and PMR [76], are available.

### 3.7. Metabolite Identification

Due to the complexity and chemical diversity of the metabolome with metabolite concentrations spread over a wide range of magnitudes, identification is still a challenge. In addition to the variety of instruments and their different operation and acquisition methods, even more programs exist for data processing, typically for a specific class of data. For example, different instrument settings used in LC-MS, such as collision energy, resolution, targeted, non-targeted, and fragmentation methods, such as CID (collision induced dissociation), HCD (higher energy collision dissociation), ETD (electron-transfer dissociation), and PQD (pulsed-Q dissociation) [77,78,79,80], can give rise to different fragments species and intensities. Hence, it is a tough task to use or build metabolite-specific spectral databases for compound identification.

One of the best, but time consuming, options is to create an instrument specific MRM method for a set of preselected metabolites. The advantage of using up to three MRMs and their MRM ion ratios, which are stable properties between transitions [81] for robust and unambiguous analysis of complex samples, was demonstrated in a screening approach of rotenone-treated HeLa cells [53]. In this study, a MRM method was developed using pure substances and the transitions had to co-elute at the same retention time with identical peak shapes. MRM ion ratios have been shown to be essential to avoid false-positive identifications in an example for inosine 5′-monophosphate [53]. Schürmann and colleagues revealed the need of a third transition as well [52]. In their study, product ions from a co-eluting interfering matrix compound were consistent with MRMs of two sebuthylazine transitions [52]. Following the European Union directive 2002/657/EC, which regulates the confirmation of suspected positive identifications, this would have resulted in a false-positive finding. To date there is an insufficient discussion about the relative importance of using certain transitions for compound identification [52]. Fragment mis-assignments can originate from impure Q1 isolation [39], co-eluting isobaric compounds, wrong database entries, or incorrect peak picking.

Due to its complex nature, non-targeted metabolomics has to be linked to advanced chemometric techniques, to reduce the data complexity into a smaller set of manageable signals [82]. Analytical methods and required improvements in non-targeted metabolomics are extensively described in a review of Alonso and colleagues [83]. A METLIN search for the exemplary parent mass 136 Da reveals 131 isobaric, but unique metabolites, many of them with very similar structures and, thus, almost similar fragment spectra. As a result, a ranked list based on similarity scores is provided and cutoff values have to be used to verify the identification. It is debatable whether this is sufficient enough and how many false-positive identifications are actually included. A big effort is being done to improve spectral databases, but the development of accurate automatic identification algorithms is still subject to the availability of an exhaustive set of reference metabolite spectra [83].

As target identification is one of the most critical steps, *in silico* target identification methods, including chemical similarity database searches, are used, such as CSNAP (Chemical Similarity Network Analysis Pulldown) [84]. Several strategies exist on how unknown peaks can be deciphered and interpreted, but validation guidelines were missing for a long time. A collection of guidelines/minimum requirements for the validation of metabolite identification, were finally conceived by the Metabolomics Standards Initiative (MSI in 2005, in order to allow data to be efficiently applied, shared and reused [85]. The initiative “COordination of Standards in MetabOlomicS” (COSMOS) is generating robust data infrastructures and exchange standards for metabolomics data and metadata [86].

## 4. Biological Interpretation of Results

At this point, a large knowledge gap exists in the translation from changes in metabolite concentration in body fluids to organ biochemistry and (molecular) physiological interpretation [20]. Existing scarce information are usually only available for specific species, organs, or body fluids and cannot be transferred from one another easily. Software tools, such as pathway or enrichment analysis are not taking that into account.

What does it actually mean, if metabolite X from a distinct pathway is found to be decreased? On one hand, downstream enzymes could have an increased activity or, on the other hand, the enzyme producing metabolite X could have a decreased activity; both can lead to a decreased level of metabolite X. Enzyme activities can be influenced by their specific product by steric inhibition/feedback-inhibition or activation, and thereby guarantee a balanced homeostasis in the cell. This strategy avoids large metabolic changes with a potential negative effect for the cell.

Many metabolites play a role in several pathways and are the product or substrate of many different enzymes or processes. Thus, it is a challenge to pinpoint an altered metabolite to a specific pathway or enzyme. Nevertheless, the pathway information may already give the correct answer, or at least a hint, for a biological question. Changes in metabolite abundances can be mapped to specific pathways, thereby providing mechanistic information of the process under study. Data derived from metabolic profiling can be complemented by genome, proteome, clinical, and environmental data, which supports the discovery of potential biomarkers that would not have been identified with targeted studies alone [87].

Metabolome profiles have the advantage to detect unknown compounds (shotgun) and alterations on a global scale (shotgun and targeted). As many metabolites usually originate from several different processes, specific methods have to be used for validation, like a knockdown of the according enzyme or isotope-labeled MFA. Many bioinformatics tools are continuously created to address several important questions in the metabolomic field, like interpreting profiling data. MetaboAnalyst (www.metaboanalyst.ca), permits a comprehensive metabolomic data analysis, visualization and interpretation, including complex statistical calculations [88]. Metabolite pathway enrichment analysis (MPEA) was designed for the visualization and biological interpretation of metabolic profiling data at the system level. The tool tests whether metabolites involved in some predefined pathways occur towards the top or bottom of a ranked query compound list [89]. Integrated Molecular Pathway Level Analysis (IMPaLA) [90] is a tool for pathway over-representation and enrichment analysis with expression- and metabolite data. This proves the importance of metabolomics, but on the downside, many of these programs get funding for only a few years and are abandoned thereafter and egress, or even become useless, as file formats and necessary accompanying software changes permanently. Furthermore, most of the programs require specific raw or input data, which are frequently not interchangeable between programs.

### Biomarker Discovery

Biomarker discovery is driven by applying new instrumentation, protocols, and software tools, in order to find novel and specific key metabolic features, which are characteristic for specific pathological conditions, diseases, or cancer. Surprisingly, the clinical breakthrough is still out of sight. Nevertheless, metabolomic key features indicative for diseases such as depression [91], schizophrenia [92,93], cardiovascular and coronary artery disease [94], diabetes [95,96], and cancers, such as liver [97], ovarian [98], and breast cancers have been reported [99]. The metabolic level variation between people, as well as within tissues and time points, is huge and dynamic. The genome, epigenome, transcriptome, and proteome states are much more stable compared to the high fluctuating metabolites. The aim in the biomarker field is to find biomarkers, which can precisely detect an early malignancy in order to achieve the best treatment effects and finally the highest survival rates for patients. So far, there were no new approved biomarkers in recent years [100,101]. The current strategy to find single biomarkers for a disease is hampered by high and dynamic fluctuations of metabolites. As every disease not only changes one metabolite, but entire metabolic pathways, we probably should search for differentially regulated pathways or metabolite classes to be the more robust biomarkers in the future.

This can be accomplished the best by an integrative approach taking many omics subdisciplines into account [102]. Thus, accurate multi-data analyses will be the key to reveal, assess, and track molecular patterns, which reflect disease-perturbed networks [102,103].

## 5. Conclusions

The metabolome, lying closest to the phenotype and the most predictive of phenotype, is of emerging interest for systems biologists. Metabolomics has a long lasting importance in the field of biomarker detection and, finally, for drug treatments. No universal instrument or method exists yet, which is capable of measuring the entire metabolome at once. Currently, we are still in the status of metabolomic profiling, intensive parallelization of different approaches and methods are transforming metabolomic profiling steadily into metabolome profiling. For a holistic view, integration of genome, transcriptome, proteome, and/or metabolome datasets can significantly enhance the insight into biological questions and molecular interactions between these disciplines. The integration of different molecular profiling data into a comprehensive entity is still challenging, especially in the bioinformatics area. Knowledge about the function of metabolites and, specifically, their multiple biological interactions are still missing. Thus, interpretation of results has to be handled with care. Our review highlights the advantages of metabolome profiles for systems biology approaches, as well as the current pitfalls, such as data interpretation and translation of results into a biologically-useful meaning.
